# The Improvement of Road Performance of Foam Asphalt Cold Recycled Mixture Based on Interface Modification

**DOI:** 10.3390/polym17141927

**Published:** 2025-07-13

**Authors:** Han Zhao, Yuheng Chen, Wenyi Zhou, Yichao Ma, Zhuo Chen, Junyan Yi

**Affiliations:** 1 School of Transportation Science and Engineering, Harbin Institute of Technology, Harbin 150090, China; 2 Jilin Traffic Planning and Design Institute, Changchun 130021, China

**Keywords:** reclaimed asphalt pavement (RAP), foam asphalt, cold recycled asphalt mixture, modifier, road performance

## Abstract

With the increasing demand for highway maintenance, enhancing the resource utilization of reclaimed asphalt pavement (RAP) has become an urgent and widely studied issue. Although foam asphalt cold recycling technology offers significant benefits in terms of resource utilization and energy saving, it still faces challenges, particularly the poor stability of foam asphalt mixtures. This study focuses on optimizing the performance of foam asphalt recycled mixtures through interface modification, aiming to promote the widespread application of foam asphalt cold recycling technology. Specifically, the research follows these steps: First, the optimal mix ratio of the recycled mixtures was determined based on the fundamental properties of foam asphalt and RAP. Then, zinc oxide, silane coupling agents, and amine anti-stripping agents were introduced to modify the recycled mixtures. At last, a series of tests were conducted to comprehensively evaluate improvements in road performance. The results indicate that the silane coupling agent enhances the low-temperature performance and fatigue. The fracture energy reached 526.71 J/m^2^. Zinc oxide improves the low-temperature cracking resistance and dry shrinkage performance. Amine anti-stripping agents have minimal impact on the low-temperature performance. The linear shrinkage was reduced by 2.6%. The results of TOPSIS indicated that silane coupling agent modification exhibits superior fatigue resistance and low-temperature performance, achieving the highest comprehensive score of 0.666. Although amine-based anti-stripping agents improve fatigue life, they are not suitable for modifying foamed asphalt mixtures due to their detrimental effects on low-temperature performance and moisture resistance.

## 1. Introduction

With the continuous expansion of highway networks, highway maintenance has become essential for enhancing pavement performance and extending service life. However, large-scale maintenance activities have generated significant amounts of reclaimed asphalt pavement (RAP), which not only consume valuable land resources but also pose serious environmental concerns [1,2,3]. With the growing emphasis on low-carbon and sustainability concepts, various industries are actively seeking more environmentally friendly approaches to reduce carbon emissions and resource consumption [4]. Cold recycling technologies have gained widespread attention and application due to their advantages, including room-temperature construction and low energy consumption [5]. Among these technologies, foam asphalt cold recycled mixtures have become a direction for RAP resource utilization, offering benefits such as energy savings, reduced asphalt mixing and transportation costs, and compatibility with both flexible and rigid pavements [6,7,8].

A foam asphalt cold recycled mixture is a sustainable pavement material composed of RAP, a small amount of new aggregate, cement, and foam asphalt as binders and molded at room temperature [9]. Theoretically, this mixture combines the asphalt’s flexibility with cement concrete’s rigidity, effectively addressing the low bearing capacity of flexible pavements and the cracking issues of rigid pavements. Foam asphalt cold recycling technology first emerged in the United States in the mid-20th century and later gained widespread adoption in countries such as South Africa, Germany, and Australia. Research on this technology has focused extensively on material selection, mix design, construction monitoring, and maintenance strategies [10,11]. However, in practical applications, challenges such as poor foam asphalt stability and high variability in RAP properties have hindered the road performance of recycled mixtures.

According to the recent studies, the amount of foam asphalt, the dosages of RAP and cement, the gradation of the mixture, and the performance of the foam asphalt affected the road performance of foam asphalt mixes [12,13]. Scholars have extensively researched foam asphalt cold recycling agents and their impact on road performance [14,15,16]. Ruckel et al. [17] found that the proportion of fine aggregates, particularly cement and mineral filler content, significantly affects the road performance of cold recycled mixtures. Similarly, Niazi and Jalili [18] reported that adding cement and lime substantially increases the strength of recycled mixtures. Graziani et al. [19] explored the fatigue resistance of foam asphalt cold recycled mixtures through tensile fatigue testing and concluded that cement significantly enhances fatigue resistance. Chen et al. [20] conducted a comprehensive evaluation of recycled mixtures with varying foam asphalt content, using the wet and dry split strength ratio, freeze–thaw split strength ratio, and fatigue life as key indicators. Their results showed that the cold recycled mixtures exhibited optimal road performance when the foam asphalt content was 3.0%. Tao et al. [21], based on rheological tests, demonstrated that foam asphalt technology significantly improves the viscoelastic properties of recycled mixtures. Additionally, Liang et al. [22] studied the impact of different RAP content levels on the performance of foam asphalt cold recycled mixtures. Their findings, based on storage stability and compatibility tests, indicated that higher RAP content negatively affects the storage stability and compatibility of the mixtures.

Although extensive research has been conducted on the road performance of foam asphalt cold recycled mixtures, with numerous findings reported, traditional foam asphalt mixtures remain limited to low-traffic asphalt pavements due to inherent performance deficiencies and are predominantly used in base layers [23]. To address these issues, incorporating modifiers into foam asphalt cold recycled mixtures has emerged as a critical strategy to enhance performance and extend service life [13]. Developing effective modification techniques has become an urgent and significant research focus in the field of road engineering. Researchers have explored using modifiers such as SBS and water-based epoxy resins to further enhance the performance of foam asphalt mixtures. However, the modified asphalt often failed to achieve satisfactory foaming performance [24].

In addition to adding modifiers to asphalt, modifying the asphalt–aggregate interface is also a commonly used method to enhance the performance of asphalt mixtures. Zhou et al. [25] employed silane coupling agents to strengthen the interfacial adhesion between reclaimed aggregates and asphalt in hot recycled mixtures, finding that the agents reduced the hydrophilicity of the aggregate surface and improved the weak interface. Yan et al. [26] developed a composite interfacial modifier that enhanced the bonding performance between plant fibers and asphalt in hot mix asphalt. Zhang et al. [27] prepared a novel anti-stripping composite containing montmorillonite and polyamide to improve the interfacial adhesion between acidic aggregates and modified asphalt. The results showed that the modified mixtures exhibited significantly improved high-temperature performance and moisture stability. Current research findings suggest that interface modification is an effective method for improving asphalt mixtures, but the selection of appropriate modifiers is crucial.

Most existing studies on interface modification have focused on hot mix asphalt, while research on cold mix asphalt—particularly foamed asphalt mixtures—remains limited. In summary, the current methods of enhancing the performance of foam asphalt are highly flawed, and systematic studies on improving their performance through different interfacial modification methods remains limited [28]. To enhance the road performance of foam asphalt cold recycled mixtures and expand their application, this study first evaluated the basic properties of foam asphalt and RAP materials, determining the optimal mix ratio. Subsequently, zinc oxide, silane coupling agents, and amine anti-stripping agents were selected from different perspectives to modify the interface of the recycled mixtures. Finally, the effectiveness of these modifiers was assessed through wet and dry split strength tests, dynamic modulus tests, semicircular bending tests, and fatigue tests. This research holds significant theoretical value in promoting the widespread application of foam asphalt cold recycling technology while also helping to mitigate the environmental pollution caused by waste pavement materials.

## 2. Materials and Methods

### 2.1. Materials

#### 2.1.1. Foam Asphalt

This study used grade 80/100 matrix asphalt and pure water to prepare foam asphalt. The basic properties of matrix asphalt are presented in Table 1. All indicators fall within the specified limits, and the performance meets the requirements for practical application. The test methods refer to the Standard Test Methods of Bitumen and Bituminous Mixtures for Highway Engineering (the Methods) [29].

**Figure 1:**
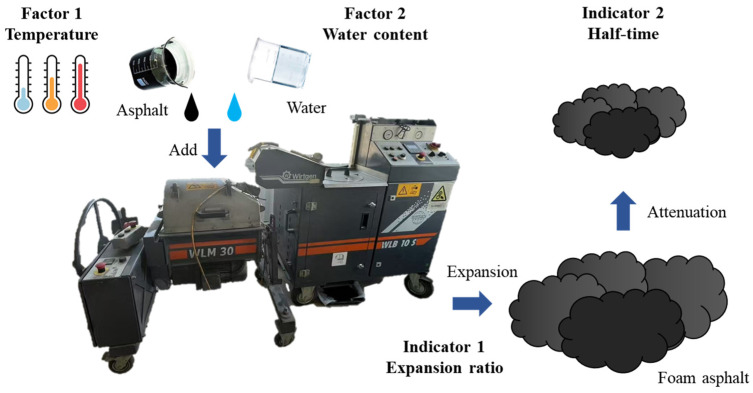
Foam asphalt foaming process and influencing factors.

The foaming process of foam asphalt and the factors affecting it are shown in Figure 1. The temperature and water content are the main factors affecting the foaming effect, and the expansion ratio and half-time are the main evaluation indexes [23].

To determine the optimal foaming temperature and water content, foam asphalt was prepared using Wirtgen foaming equipment at temperatures of 150 °C, 160 °C, and 170 °C, with water contents of 1%, 2%, 3%, and 4%. The expansion ratio and half-time of the produced foam asphalt were measured, and the results are summarized in Table 2.

The results of the expansion ratio test showed that the temperature effect was significant. At 160 °C, the foam bitumen has the highest expansion ratio. The results of the half-time test showed that there was little difference between the three temperatures. A higher expansion ratio indicates that more foam is generated by the asphalt, resulting in a larger surface area that more effectively coats the aggregates, thereby enhancing adhesion and diffusion. Therefore, 160 °C was selected as the foaming temperature.

In practical engineering, an excessively short half-life leads to rapid foam collapse, which may result in insufficient mixing and increased construction difficulty. Conversely, a half-life that is too long may indicate excessive moisture retention, which can potentially compromise early strength. A half-life of 8–15 s is considered ideal. A low expansion ratio indicates poor foaming performance, which is unfavorable for mixing. On the other hand, an excessively high expansion ratio may lead to incomplete reactions between the asphalt and water or introduce unnecessary moisture. An expansion ratio of 10 or greater is generally considered optimal. Based on the requirements of the Technical Specifications for Highway Asphalt Pavement Recycling [30], the expansion ratio is not less than 10 times, and the half-time is not less than 8 s. According to the test results, the minimum water content that meets the allowable expansion ratio is 1.7%, and the maximum water content that meets the allowable half-time is 3.3%. Taking the average of the two as the best water content of 2.5%, then, the expansion ratio is 14 with a half-time of 15 s, in line with the specification requirements. Therefore, the optimal foaming temperature and foaming water content were determined to be 160 °C and 2.5%.

#### 2.1.2. RAP

The RAP material used in this study was sourced from a repaired section of the Changyu Expressway. To assess its suitability for foam asphalt cold recycled mixtures, the basic properties of the aged aggregates and asphalt within the RAP material were evaluated. The properties of the aged aggregates were tested following the Methods [30], and the results are presented in Table 3.

**Figure 2:**
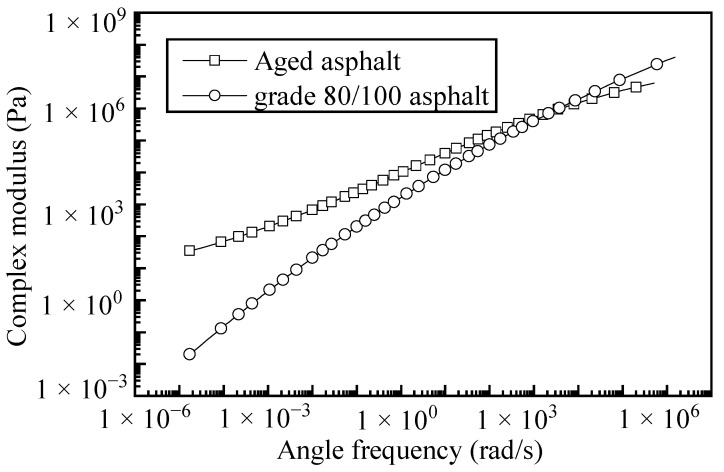
Dynamic modulus master curve of aged asphalt.

As shown in Table 3, the properties of the RAP, both before and after extraction, comply with the required specifications. The aged asphalt content in the RAP was determined to be 4.2% by measuring the mass of the aged aggregate before and after extraction. The basic properties of the aged asphalt were tested according to the Methods [31], and the results are presented in Table 4. In addition to the basic properties, the dynamic modulus master curve of aged asphalt was tested using a dynamic shear rheometer, and the results are shown in Figure 2.

The changes in both the basic properties and the dynamic modulus master curves indicate that the asphalt in the RAP is severely aged. At the same temperature, the G* of the aged asphalt was greater than that of 90# matrix asphalt, indicating that its viscoelastic properties were severely degraded and the aging of the asphalt was high [32].

#### 2.1.3. Modifiers

**Figure 3:**
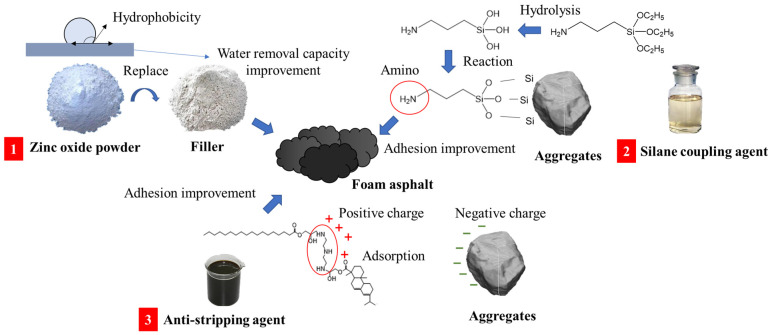
Modification mechanisms of modifiers.

To enhance the road performance of foam asphalt recycled mixtures, this study investigates three interface modification approaches. These modifications aim to improve the interfacial properties and optimize the overall performance of the mixtures. The optimal dosage of the three modified materials was determined through a pretest, and the modification mechanism of different modifiers is shown in Figure 3.

1. Filler Replacement: Zinc oxide powder was used as a substitute for the mineral filler in the recycled asphalt mixture, with a replacement dosage of 5%. Replacing part of the mineral powder with zinc oxide powder improves the hydrophobicity of the asphalt mastic, which in turn enhances the water removal capacity of the entire foam asphalt mix [33].
2. Surface Modification of RAP Material: A silane coupling agent–ethanol–water solution was applied to modify the surface of the RAP material. The solution dosage was 2% of the total aggregate mass, with a silane coupling agent–ethanol–water ratio of 1:5:10. While the coupling agent reacts chemically with the aggregate, its organic functional groups are very compatible with the asphalt, thus improving the bond strength between the aggregate and the asphalt [34].
3. Foam Asphalt Modification: An amine-based anti-stripping agent was incorporated into the foam asphalt with a dosage of 0.3%. Anti-stripping agents contain alkaline groups and are soluble in asphalt. The polar cationic terminals are chemisorbed by ionic bonding with the aggregate surface, thus improving the bonding strength of the aggregate to the asphalt [35].

### 2.2. Preparation Methods

#### 2.2.1. Mixing Ratio Design

**Figure 4:**
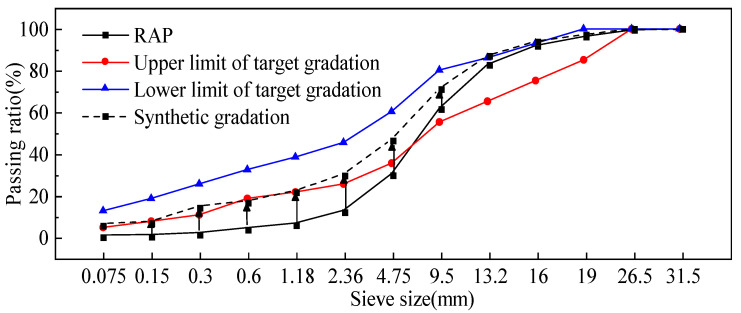
Target gradation and gradation of RAP.

According to the Technical Specification for Highway Asphalt Pavement Recycling [24], this study discarded old aggregates with a sieve aperture > 19 mm from the original RAP material and selected a medium-grain gradation as the target. The original RAP gradation and the target gradation are shown in Figure 4.

Fine aggregate and mineral powder (≤4.75 mm) were added. This adjustment ensures that the final gradation meets the specification requirements and provides the necessary strength for the mixture. In this study, the Technical Specification for Highway Asphalt Pavement Recycling [29] was used as a reference to determine the material composition of the foam asphalt cold recycled mixture. The selected mix proportions are as follows: cement: 1.5%; mineral powder: 4.5%; fine aggregate: 4.0%; and RAP material: 90%. The synthetic gradation results for the foam asphalt cold recycled mixture are presented in Figure 4.

#### 2.2.2. Preparation Method

**Figure 5:**
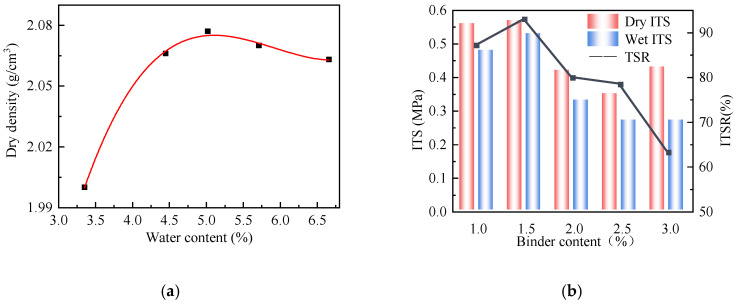
Optimum water and binder content test results. (**a**) Compaction test. (**b**) Wet and dry ITS tests.

This study conducted the inorganic binding material stabilization test according to the Test Methods of Materials Stabilized with Inorganic Binders for Highway Engineering [36] to determine the optimal mixing water content for preparing the foam asphalt cold recycled mixture. To determine the optimal foam asphalt dosage, foam asphalt cold recycled mixtures were prepared using 1.5%, 2.0%, 2.5%, 3.0%, and 3.5% foam asphalt. Marshall test specimens were fabricated according to the Standard Test Methods of Bitumen and Bituminous Mixtures for Highway Engineering [31], and wet and dry indirect tensile strength (ITS) tests were conducted. The test results are shown in Figure 5.

The results in Figure 5a show that the mixing water content required was 5.0%. The maximum dry density of the mix was 2.075 g/cm^3^. The strength of foamed asphalt cold recycled mixtures depends on aggregate gradation, asphalt, and cement [6]. At the early stage, the cohesive–adhesive system formed by cement hydration and foamed asphalt is not yet fully developed. Excessive compaction may cause rapid moisture loss, which disrupts the foam structure and adversely affects early strength development. Considering practical engineering applications, where 80% compaction is often used as the reference for determining the optimal water content, 4.0% was ultimately selected as the best mixing water content for preparing foam asphalt cold recycled mixtures.

The results in Figure 5b indicate that the dry ITS initially increased, then decreased, and finally increased again as the foam asphalt dosage increased. The wet ITS increased at first and then decreased. The maximum dry and wet ITS ratio (ITSR) was observed at a 1.5% foam asphalt dosage. At the given dosage, the foamed asphalt can effectively coat fine aggregates and form a stable skeletal structure, while maintaining good adhesion under wet conditions. Based on these findings, 1.5% was selected as the optimal foam asphalt dosage for preparing foam asphalt cold recycled mixtures. At this dosage, the dry ITS reached 0.57 MPa, and the TSR was 92.98%, meeting the requirements of dry ITS > 0.4 MPa and TSR > 80% in the Technical Specification for Construction of Highway Asphalt Pavements (JTG F40-2023) (the Specification) [37]. In summary, 1.5% foamed asphalt combined with 4.0% water represents the optimal formulation for the current material system, balancing early strength, moisture stability, mixing workability, and long-term durability. Either excessive or insufficient binder, as well as overly high or low moisture content, can disrupt the microstructure and overall performance of foamed asphalt cold recycled mixtures, ultimately reducing their long-term service life.

After determining the optimal water content and binder dosage, four types of recycled mixtures were prepared: ordinary foam asphalt cold recycled mixtures, foam asphalt mixtures with zinc oxide, mixtures containing a silane coupling agent, and those with an amine anti-stripping agent. The specimens were then placed in an oven at 40 °C for 72 h, stored at room temperature for 12 h, de-molded, and tested for road performance.

### 2.3. Performance Evaluation Methods

#### 2.3.1. Basic Performance

(1) Wet and dry ITS test

This study performed wet and dry ITS tests on the de-molded foam asphalt mixture specimens, referring to AASHTO T 283 [38]. The dry ITS was measured by soaking the specimens in a water bath at 15 ± 0.5 °C for 2 h, followed by a tensile test. The wet ITS was determined by soaking the specimens in a water bath at 25 ± 0.5 °C for 22 h, then transferring them to a water bath at 15 ± 0.5 °C for 2 h, and performing the tensile test. The ratio of the wet ITS to the dry ITS was used to calculate the ITSR for different foam asphalt mixtures. All the performance tests were carried out five times, the average value was taken as the test result, and the standard deviation was calculated to plot the error bars.

(2) Semicircle bending test

This study used the semicircular bending test to evaluate the low-temperature cracking resistance of different foam asphalt mixtures, referring to ASSHTO T 393 [39]. A universal testing machine was used to apply loading to semicircular bending and pulling specimens with a diameter of 100 mm, a thickness of 25 mm, and a slit length of 15 mm (with the slit perpendicular to the bottom of the semicircular specimen). The specimens were held at −30 °C for 4 h, then preloaded to ensure contact between the indenter and the specimen. The specimen was then loaded at a 0.02 mm/min rate until failure. The peak fracture load, fracture energy, and fracture toughness were measured to assess the low-temperature cracking performance of the foam asphalt mixtures.

(3) Drying shrinkage test

With reference to the Standard test method for drying shrinkage of mortar (JC/T 603-2004) [40], a drying shrinkage test was conducted on different types of foam asphalt mixtures. In this study, the dry shrinkage test was carried out in a 20 °C environment. The water loss and shrinkage rate of foam asphalt concrete were calculated to evaluate the dry shrinkage performance of different types of foam asphalt mixes.

#### 2.3.2. Fatigue Test

(1) Three-Point Bending Fatigue Test

In this study, a three-point bending fatigue test was conducted using a universal testing machine on the asphalt mixture, referring to JTG E20 T 0715 [29]. The test loading rate was 50 mm/min. The temperature was 25 °C and the stress ratios were 0.5, 0.7, and 0.9. In the stress control mode, the relationship between the number of loading cycles and the loading stress ratio was calculated as Equation (1).

(1) $$N_{f}=k{\left(\frac{1}{\sigma }\right)}^{n}$$

where *N_f_* is the fatigue life (times), *σ* is the stress ratio, and *k* and *n* are the fatigue test fitting parameters.

(2) Indirect Tensile Test

This study used a universal testing machine to perform the indirect tensile test on asphalt mixture cylinder specimens, referring to JTG E20 T 0716 [29]. The upper and lower compression strip widths were set to 12.7 mm, and the test loading rate was 50 mm/min. The temperature was 25 °C and the stress ratios were 0.5, 0.7, and 0.9.

#### 2.3.3. Dynamic Modulus Test

Referring to ASSHTO T 378 [41], a universal testing machine was used to apply sinusoidal loads of 0.1 Hz, 0.5 Hz, 1 Hz, 5 Hz, 10 Hz, and 25 Hz to the foam asphalt mixtures with a diameter and height of 100 mm at temperatures of −10 °C, 5 °C, 20 °C, 35 °C, and 50 °C. The loads at different temperatures were 2000 kPa, 1000 kPa, 500 kPa, 200 kPa, and 50 kPa. These loads simulate the real driving forces that pavements experience during service. The complex modulus and phase angle obtained from these dynamic modulus tests were used to evaluate the mechanical properties of the asphalt mixtures at different temperatures.

### 2.4. TOPSIS Analysis Method

Since the macroscopic performances represented by each index in the tests were quite different for each modification method, TOPSIS was used to analyze the optimal method [42]. In this study, the representative ITSR, fracture toughness, linear shrinkage, and three-point bending fatigue life at a 0.7 stress ratio were selected as the performance evaluation indexes. Since the moisture sensitivity, low-temperature cracking resistance, drying shrinkage performance, and fatigue resistance represented by the four indexes are directly related to the road performance of the mix, the four indexes are regarded as equally weighted indexes. The z-score method was used for standardization.

## 3. Results and Discussion

### 3.1. Basic Performance

#### 3.1.1. Wet and Dry ITS Test

**Figure 6:**
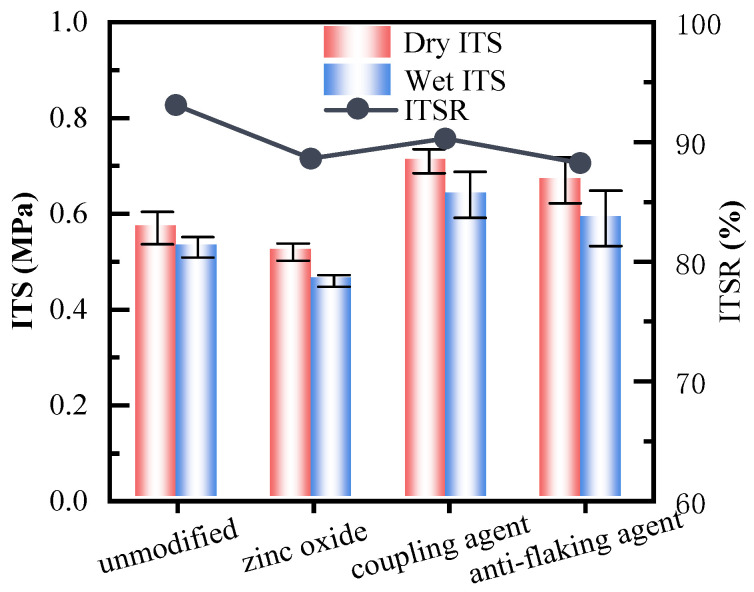
Dry and wet ITSs of different foam asphalt cold recycled mixtures.

The dry and wet ITSs and their ITSR serve as effective indicators for evaluating the water damage resistance of foam asphalt mixtures. Figure 6 presents the dry and wet ITSs of different foam asphalt cold recycled mixtures and their corresponding strength ratios.

As shown in Figure 6, all the foam asphalt cold recycled mixtures meet the requirements of a dry splitting strength > 0.4 MPa and TSR > 80% in the Specifications. However, the effect of different modifiers on water damage resistance varied. The addition of zinc oxide had minimal impact on improving the dry and wet splitting strengths. In contrast, incorporating a silane coupling agent enhanced both strengths by approximately 10% compared to unmodified foam asphalt mixtures. Adding an amine anti-stripping agent resulted in an even more significant improvement, with an increase of roughly 20%. These findings demonstrate that silane coupling agents and amine anti-stripping agents significantly enhance this mechanical property of foam asphalt mixtures. Both methods enhanced the adhesion between asphalt and aggregates through different interfacial modification mechanisms. The silane coupling agent, due to its bifunctional structure, formed stable covalent bonds on the surface of the mineral aggregates and interacted with the polar components in the asphalt, thereby forming a robust organic–inorganic interfacial layer. The amine-based anti-stripping agent neutralized surface charge differences on aggregates, improving the wetting and adhesion of the asphalt to acidic or hydrophilic aggregates, and significantly enhancing its resistance to stripping and moisture damage [34,35].

After modification with the three agents, the ITSR showed varying degrees of reduction. The reduction was most pronounced with zinc oxide, but the decrease did not exceed 5%, and the ITSR remained above 85%. Therefore, none of the three modifiers had a significant effect on moisture sensitivity.

#### 3.1.2. Semicircle Bending Test

**Figure 7:**
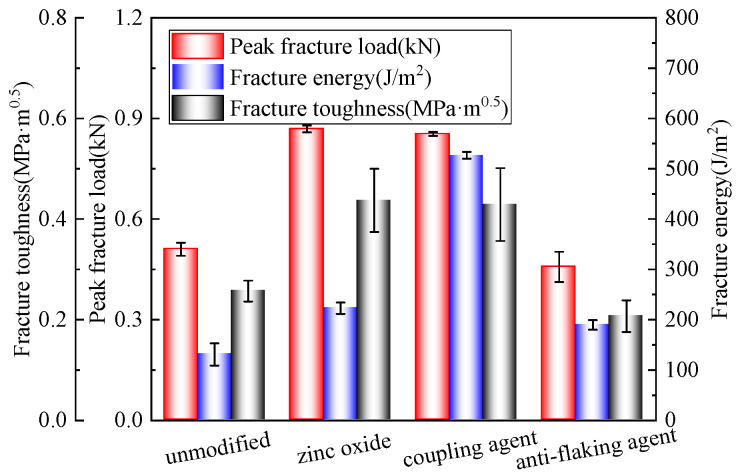
Semicircle bending test results.

Figure 7 presents different foam asphalt cold recycled mixtures’ peak fracture load, fracture energy, and fracture toughness.

The test results indicate that different modifiers had significant effects on the fracture properties of the mixes. Compared with the unmodified mixes, the zinc oxide modification showed improvements of about 70.6%, 70.0%, and 69.2% in the peak fracture load, fracture energy, and fracture toughness, respectively, and exhibited excellent fracture resistance. Although the silane coupling agent modification showed slightly lower peak fracture load and toughness enhancement (66.7% and 65.4%), the fracture energy was substantially increased by about 301.7%, demonstrating an excellent ability to absorb energy after crack extension. In contrast, the modification of the anti-stripping agent improved the fracture energy (44.6%), but the peak fracture load and toughness were reduced, indicating that it may weaken the fracture resistance.

Existing studies have shown that, for a specimen with a diameter of 100 mm, a fracture energy greater than 400 J/m^2^ implies that the material has excellent low-temperature crack resistance [30]. The addition of a silane coupling agent significantly improved the low-temperature performance of the foam asphalt mixture, with a fracture energy of 526.71 J/m^2^. This is because, in the foamed asphalt cold recycling mixtures, silane can react with hydroxyl groups on the aggregate surface to form stable siloxane bonds, while its organic side chains interact with polar components in the foamed asphalt, significantly enhancing the adhesion and interfacial stability between the aggregate and asphalt [34].

#### 3.1.3. Dying Shrinkage Test

**Figure 8:**
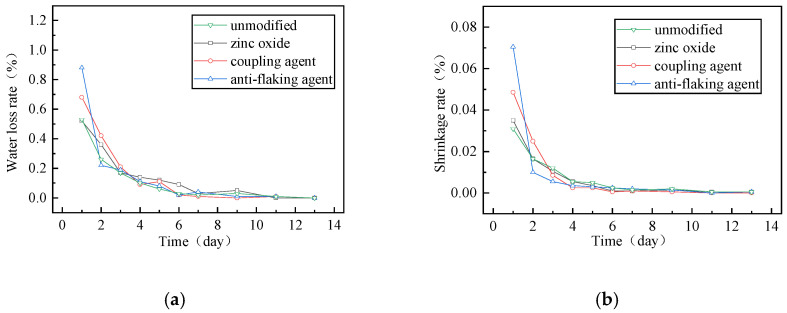
Drying shrinkage test results (**a**) Water loss rate. (**b**) shrinkage rate.

The results of the drying shrinkage test are shown in Figure 8.

Based on the drying shrinkage rate and water loss rate, it was observed that at 20 °C, all mixtures lost 95% of their water within the first five days. The mixture containing an anti-stripping agent exhibited a water loss rate twice that of the ordinary foam asphalt mixture, while the coupling agent-modified mixture showed a 50% increase. In contrast, the zinc oxide-modified mixture demonstrated similar water loss and dry shrinkage rates to the ordinary foam asphalt mixture, with negligible differences observed among different dosages of the same additive.

The cumulative linear shrinkage values for the four mixtures were 0.078%, 0.076%, 0.089%, and 0.108%, respectively. The zinc oxide-modified mixture exhibited the lowest shrinkage (0.076%), indicating the best shrinkage resistance. This is because zinc oxide powder improves the dewatering capacity of the foamed asphalt mixture [33]. The coupling agent and anti-stripping agent accelerated water evaporation, resulting in increased linear shrinkage by 14% and 38.5%, respectively.

### 3.2. Fatigue Performance

#### 3.2.1. Three-Point Bending Fatigue Test

**Figure 9:**
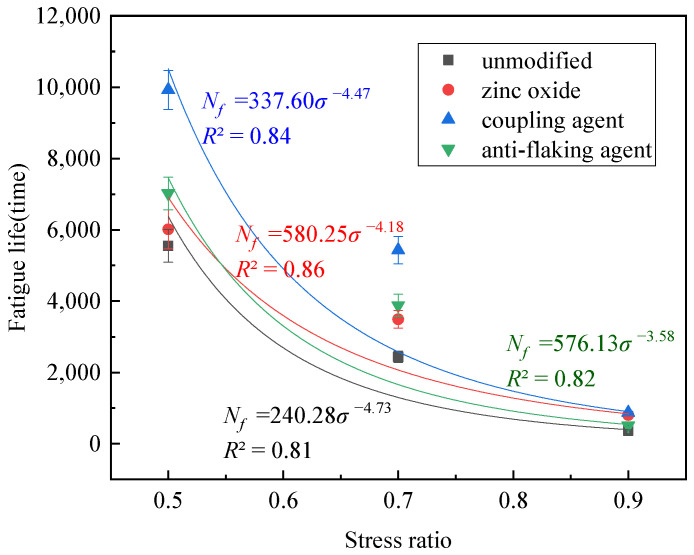
Bending ITS and fatigue life of different foam asphalt cold recycled mixes.

The strength and fatigue life of the different foam asphalt cold recycled mixes are shown in Table 5 and Figure 9.

Adding modifiers will improve the strength and fatigue life of foam asphalt recycled mixtures, improving the fatigue resistance of foam asphalt recycled mixtures. Adding a silane coupling agent improves the fatigue resistance of foam asphalt reclaimed mixtures to the best extent, followed by adding an amine anti-scalping agent. In contrast, the presence of zinc oxide improves the fatigue resistance of reclaimed mixtures to a lesser extent. The strength of the three-point bending test is mainly dependent on the bond between the aggregate and the binder [43]. The significant increase in strength with no change in binder implies that the modifier effectively improves the interfacial adhesion between the aggregate and binder. The obtained fitting parameters are shown in Table 6.

In general, in the fatigue test fitting parameters, the larger the *n* value, the stronger the sensitivity of the material to the change of stress level, and the larger the *k* value, the higher the fatigue life of the material [44]. The *k*-values of the four mixes are in the following order: unmodified < coupling agent < anti-stripping agent < zinc oxide. Additionally, the *n*-values are in the following order: unmodified > anti-stripping agent > zinc oxide > coupling agent. Although the *k* and *n* values of different types of modifiers are not in the same order, they are all better than unmodified, which means that the modifiers can effectively improve the fatigue life of the mixture and reduce its sensitivity.

#### 3.2.2. Indirect Tensile Test

**Figure 10:**
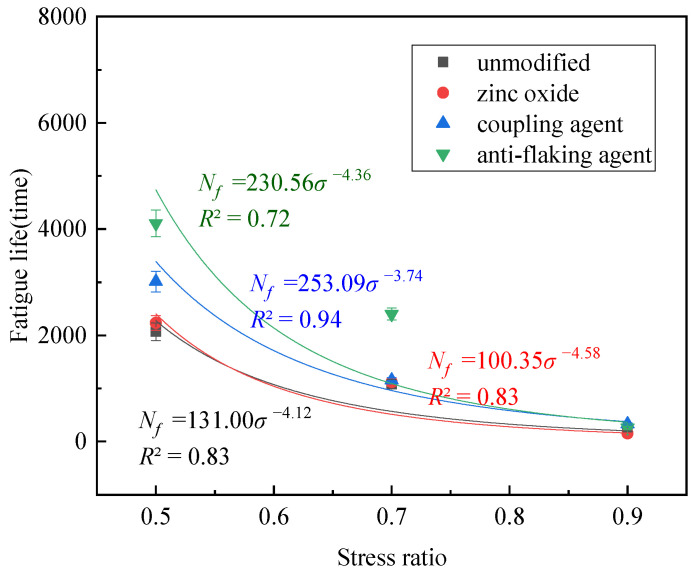
Splitting strength and fatigue life of different foam asphalt cold recycled mixes.

The splitting strength of different foam asphalt cold recycled mixes and the fatigue life are shown in Table 7 and Figure 10.

The results show that the presence of the silane coupling agent and amine anti-stripping agent significantly improve the splitting strength and fatigue life of reclaimed mixtures compared with that of the foam asphalt cold reclaimed mixtures with no modifier added, and the amine anti-stripping agent improves the anti-fatigue performance of reclaimed mixtures to a better extent. This is because the silane coupling agent forms a robust organic–inorganic interface, while the amine-based anti-stripping agent enhances the adhesion between the asphalt and aggregate [34,35]. However, the addition of zinc oxide deteriorated the splitting strength of the recycled mixes. The results of the indirect tensile test differ from those of the three-point bending test due to variations in specimen geometry and loading conditions. Since the three-point bending test more accurately reflects the effects of interfacial modification, its results are used for performance comparison. The obtained fitting parameters are shown in Table 8.

The *k*-values for the four mixtures follow the order zinc oxide < unmodified < anti-stripping agent < coupling agent, while the *n*-values follow the order zinc oxide > anti-stripping agent > unmodified > coupling agent. The fitting coefficients align with the fatigue life results, indicating that the addition of zinc oxide reduces the fatigue life under indirect tensile conditions.

### 3.3. Dynamic Modulus

**Figure 11:**
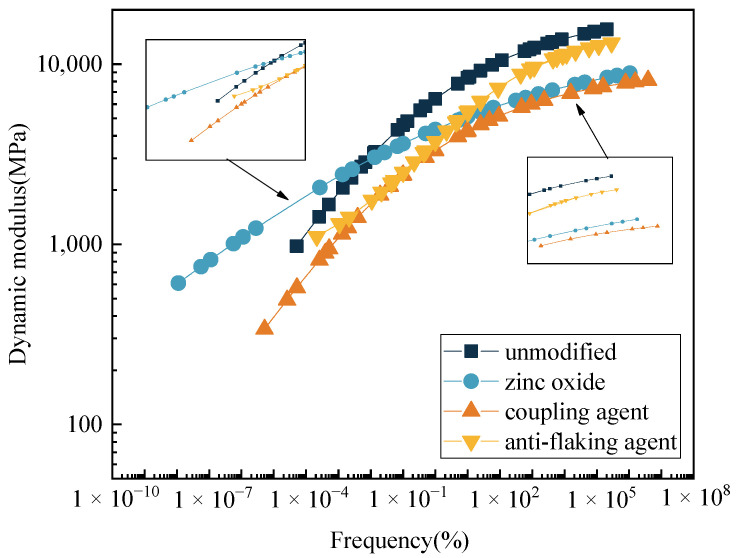
Dynamic modulus main curve at 20 °C.

Based on the time–temperature equivalence principle, the fitted dynamic modulus curve at 20 °C is shown in Figure 11.

The high-frequency curve in the dynamic modulus corresponds to the low-temperature performance, while the low-frequency curve represents the high-temperature performance. A lower modulus at high frequency indicates better low-temperature performance, and a higher modulus at low frequency indicates improved high-temperature performance [44]. Under the high-frequency condition, the modulus follows the order unmodified > anti-stripping agent > zinc oxide > coupling agent. Under the low-frequency condition, the order is zinc oxide > unmodified > anti-stripping agent > coupling agent. The modification effects of the silane coupling agent and zinc oxide on the low-temperature performance are significant, and this result is consistent with the results of the performance experiment. At 20 °C and 10 Hz, the dynamic modulus values of the four mixtures were 8389 MPa, 5107 MPa, 4243 MPa, and 5423 MPa, respectively. The addition of modifiers significantly reduced the dynamic modulus, which may adversely affect the deformation resistance of the foamed asphalt mixtures.

### 3.4. Determination of the Optimal Modification Method Based on the TOPSIS Approach

**Figure 12:**
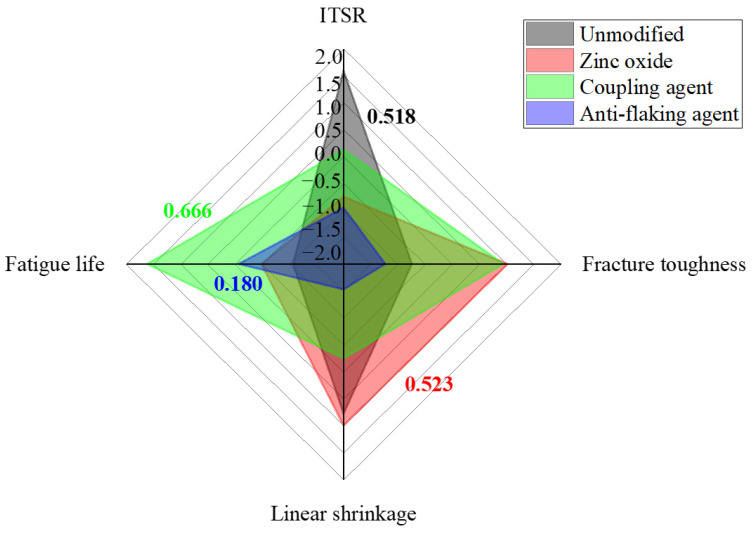
TOPSIS analysis results.

The results of the TOPSIS analysis are presented in Figure 12.

The analysis results indicated that the silane coupling agent modification exhibited superior fatigue resistance and low-temperature performance, achieving the highest overall rating of 0.666. Zinc oxide modification improved the low-temperature performance and dry shrinkage resistance. Although it negatively impacted moisture sensitivity, its overall performance surpassed that of the unmodified mixture, with a rating of 0.523. Despite enhancing the fatigue life, the anti-stripping agent demonstrated weaknesses in other performance indicators, rendering it unsuitable for foam asphalt mixture modification.

## 4. Conclusions

This study evaluated the effects of three different modifiers—a silane coupling agent, amine anti-stripping agent, and zinc oxide—on the performance of foam asphalt reclaimed mixtures. The results demonstrated that both the silane coupling agent and the amine anti-stripping agent significantly improved the dry and wet indirect tensile strengths (ITSs) of the mixtures. Both agents improve the adhesion between the asphalt and aggregates through covalent bonding and neutralization of surface charge differences, respectively. In contrast, zinc oxide had little impact on ITS performance. Notably, none of the three modifiers showed a clear improvement in moisture sensitivity, indicating that further enhancements may be required to improve resistance to water.

In terms of fracture resistance, zinc oxide was the most effective modifier, contributing to the highest peak fracture load, fracture energy, and overall toughness. This is because zinc oxide enhances the moisture evaporation capacity of foamed asphalt mixtures during the mixing, compaction, and curing stages, thereby improving early dewatering efficiency and later volumetric stability. The silane coupling agent exhibited superior energy absorption capacity, whereas the amine anti-stripping agent, despite enhancing fracture energy, resulted in a decline in both peak load and toughness. These findings highlight that each modifier influences different aspects of the fracture process and should be selected based on the specific performance priorities of the mixture.

Zinc oxide also demonstrated the strongest resistance to shrinkage, exhibiting the lowest linear shrinkage rate among all tested samples. In contrast, silane coupling agents and amine-based anti-stripping agents lack moisture evaporation capability, resulting in increased dry shrinkage after curing. Despite these differences, all three modifiers contributed to improvements in fatigue performance, with the silane coupling agent showing the most substantial enhancement in fatigue life.

Overall, the silane coupling agent offered the most balanced modification effect by simultaneously improving the strength, fracture energy, fatigue resistance, and low-temperature performance. While zinc oxide enhanced the fracture behavior and shrinkage resistance, its lack of effect on the ITS and moisture sensitivity limits its standalone applicability. The amine anti-stripping agent, although beneficial in fatigue resistance, showed trade-offs in other mechanical properties, suggesting it may be better suited as a secondary or complementary additive in foam asphalt mixture design.

## Figures and Tables

**Table 1:** Basic properties of No. 90 matrix asphalt.

Technical Indicators	Result	Requirements	Test Method
Needle penetration (25 °C)/0.1 mm	85.2	80~100	T 0604
Softening point/°C	53	>45	T 0606
Elongation (15 °C)/cm	>100	>100	T 0605

**Table 2:** Expansion ratio and half-time of different foam bitumen.

Amount of Foaming Water (%)	150 °C		160 °C		170 °C	
	Expansion Ratio (Time)	Half-Time (s)	Expansion Ratio (Time)	Half-Time (s)	Expansion Ratio (Time)	Half-Time (s)
1	7	30.7	6.7	38.7	6.7	27.4
2	10	21.7	10.7	21.3	10.7	17.3
3	12.6	14	17.3	10.8	14.3	10
4	17.3	8.6	22.7	8.6	17	5.6

**Table 3:** Basic properties of aggregate in RAP.

Materials	Test Item	Result	Requirement
RAP before extraction	Moisture content (%)	0.17	≤3
	Maximum particle size (mm)	26.50	≤26.5
RAP after extraction	Density (g/m^3^)	2.74	≥2.45
	Crushing value (%)	17.30	≤30
	Needle flake content (%)	8.90	≤15

**Table 4:** Basic properties of aged asphalt in RAP.

Asphalt Type	Needle Penetration (0.1 mm)	Softening Point (°C)	Elongation (15 °C, mm)	Dynamic Viscosity (60 °C, Pa·s)
Aged asphalt	57.1	64.9	14.6	6014.5
grade 80/100 asphalt	85.2	53	>100	160

**Table 5:** Results of the split test.

Type	Maximum Load (kN)	Strength (MPa)
Unmodified	0.6	0.51
Zinc oxide	4.6	3.68
Coupling agent	6.1	4.88
Anti-stripping agent	6.5	5.2

**Table 6:** Fitting results of the three-point bending fatigue test.

Type	Fitting Parameters	
	*k*	*n*
Unmodified	240.28	4.73
Zinc oxide	580.25	4.18
Coupling agent	337.60	4.47
Anti-stripping agent	576.13	3.58

**Table 7:** Results of the bending test.

Type	Maximum Load (kN)	Strength (MPa)
Unmodified	4.9	0.48
Zinc oxide	4.8	0.47
Coupling agent	6.4	0.63
Anti-stripping agent	6.0	0.60

**Table 8:** Results of the splitting test.

Type	Fitting Parameters	
	*k*	*n*
Unmodified	131.00	4.12
Zinc oxide	100.35	4.58
Coupling agent	253.09	3.74
Anti-stripping agent	230.58	4.36

## Data Availability

Some or all of the data, models, or code that support the findings of this study are available from the corresponding author upon reasonable request.
